# Should I stay or should I go (to transplant)? Managing insufficient responses to induction in multiple myeloma

**DOI:** 10.1038/s41408-023-00864-0

**Published:** 2023-05-30

**Authors:** Rahul Banerjee, Louis Williams, Joseph R. Mikhael

**Affiliations:** 1 Division of Medical Oncology, Department of Medicine, University of Washington, Seattle, WA USA; 2 Clinical Research Division, Fred Hutchinson Cancer Center, Seattle, WA USA; 3 Taussig Cancer Institute, Cleveland Clinic, Cleveland, OH USA; 4 Translational Genomics Research Institute (TGen), City of Hope, Phoenix, AZ USA

**Keywords:** Myeloma, Stem-cell therapies

In their 1980s classic “Should I stay or should I go,” the Clash rock band ponders whether to continue an imperfect relationship or to move on. Autologous stem cell transplantation (ASCT) for multiple myeloma (MM), another product largely of the 1980s [1], sometimes poses the same dilemma decades later for patients with potentially insufficient responses to pre-ASCT induction therapy. The historical rationale for moving to ASCT after 4–6 cycles of induction is rooted in concerns about the toxicities of induction (dating back to the days of anthracycline-based therapy) or of impaired stem cell yield after prolonged lenalidomide exposure. For patients in the modern era who have achieved no better than a partial response (PR) with induction therapy, should we stay in this induction phase or should we go directly to ASCT?

**Fig. 1:**
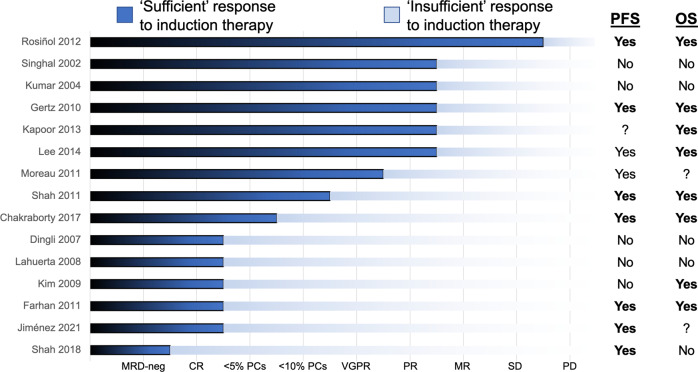
Responses to induction therapy in MM. For each study, the PFS and OS columns state whether achievement of the response deemed to be ‘sufficient’ was associated with any benefit by any statistical method; question marks mean that the specific endpoint was not investigated. ASCT autologous stem cell transplantation, CR complete response, MM multiple myeloma, MR minimal response, MRD-neg measurable residual disease negativity, OS overall survival, PCs plasma cells on bone marrow biopsy, PD progressive disease, PFS progression-free survival, SD stable disease, VGPR very good partial response.

While retrospective studies (Fig. 1) have generally shown that deeper pre-ASCT responses are associated with improved progression-free survival (PFS) after transplantation [2–16], substantial variation in induction regimens and definitions of ‘sufficient’ responses preclude any formal meta-analysis. Two older studies (Table 1) have reached opposite conclusions regarding second-line treatment intensification in patients with pre-ASCT responses to induction therapy deemed to be insufficient. In a registry-based study of patients with a minimal response (MR) or less treated between 1995 and 2010, Vij and colleagues found that second-line therapy deepened responses but did not improve PFS [17]. Conversely, in a randomized study of second-line CyBorD (cyclophosphamide, bortezomib, dexamethasone) versus proceeding to ASCT in patients with ≤PR treated between 2010 and 2016, Jackson and colleagues found that salvage induction therapy improved PFS [18]. Given that neither study routinely incorporated modern triplet regimens containing both proteasome inhibitors (PIs) and immunomodulatory imide drugs (IMIDs), how should we approach this situation in 2023?

**Table 1:** Studies of pre-ASCT treatment intensification in MM.

Study	Methods	Initial response	Intensified cohort	Other cohort	Outcome
Vij 2015 [17]	Retrospective CIBMTR registry	≤MR to a PI- or IMID-containing regimen	Second-line therapy leading into ASCT	Direct transition to ASCT	No difference in PFS or OS with addition of second-line therapy
Jackson 2019 [18]	Prospective randomized trial	≤PR to CTd or CRd	Second-line CyBorD leading into ASCT	Direct transition to ASCT	Increased PFS, but no increased OS, with additional CyBorD

*ASCT* autologous stem cell transplantation, *CIBMTR* Center for International Blood and Marrow Transplant Research, *CRd* cyclophosphamide/lenalidomide/dexamethasone, *CTd* cyclophosphamide/thalidomide/dexamethasone, *CyBorD* cyclophosphamide/bortezomib/dexamethasone, *MM* multiple myeloma, *MR* minimal response, *OS* overall survival, *PD* progressive disease, *PR* partial response, *PFS* progression-free survival, *SD* stable disease.

For patients who achieve ≤PR after 4–6 cycles of first-line induction, three biological rationales might prompt the initiation of second-line therapy with drugs like carfilzomib or pomalidomide. Firstly, although data are lacking in the modern era of measurable residual disease (MRD) testing, high-dose melphalan likely induces no higher than a 4–5 log reduction in tumor cells in patients who remain MRD positive after induction (extrapolating from Myeloma IX trial data using an MRD sensitivity of 10^−4^ cells) [19]. Given that patients with ≤PR have higher tumor burden and that deeper MRD negativity is associated with more durable responses, it follows that reducing tumor burden by all means may help maximize the ‘mileage’ of transplantation thereafter [19, 20]. As a second rationale, high-dose melphalan is mutagenic toward surviving tumor cells [21, 22]. This, in turn, may favor lowering the denominator of susceptible tumor cells beforehand. Finally, circulating PCs during collection and autograft contamination—both of which are less common with deeper responses to induction—may be associated with inadequate stem cell collection or worsened outcomes [23, 24]. If one assumes these principles to be true for every patient, then a response-based approach to induction rather than a cycle-based approach may logically lead to longer PFS and less aggressive relapses.

It is also important to note that prolonging the induction phase of therapy to ensure at least a very good partial response (VGPR) may improve the safety and feasibility of ASCT in select cases. For patients with high tumor burden and a steady response to each cycle of induction—e.g., a patient with biopsy-proven cast nephropathy and involved serum-free light chains which remain elevated even after a 50% reduction—continuing the same regimen for a few additional cycles may be reasonable to maximize pre-ASCT renal function. For patients with concurrent AL amyloidosis, changing induction therapies to induce a cardiac response may allow a previously ineligible patient to be considered for ASCT. This same principle may also apply to disease-related comorbidities such as pain and frailty, where better disease control may improve functional status to the point where transplantation becomes feasible.

However, proceeding directly to transplantation after a fixed number of cycles of induction may be the most evidence-based approach to frontline therapy in MM. In both the IFM-2009 and DETERMINATION Phase 3 randomized trials, the upfront ASCT arm moved directly to transplantation after a fixed number of cycles of induction regardless of response achieved (with the caveat that some patients with refractory disease may have withdrawn from the study) [25, 26]. Similarly, in the Phase 3 BMT-CTN 0702 trial of different ASCT approaches, half of the patients had ≤PR at study registration [27]. Many of these trials employed post-ASCT consolidation therapy, which may be a valuable tool if neither induction nor ASCT yields sufficiently deep responses. If anything, strategies like post-ASCT consolidation or multi-drug maintenance are more established strategies to manage risk in myeloma compared to a second-line pre-ASCT therapy. And although salvage CyBorD was shown to prolong PFS in a sub-randomization of the Myeloma XI trial [18], many patients diagnosed today will have access to more modern therapies in both the first and second lines.

Possibly the biggest argument in favor of moving directly to ASCT after a time-limited length of induction is the role of transplantation as the ‘equalizer’ of treatments [28]. High-dose melphalan works regardless of country, insurance type, or availability of frontline CD38-directed monoclonal antibodies. Several of the studies described in Fig. 1 have shown an association between PFS and deeper responses to induction [2, 5, 9, 14]. However, this may represent the confounding effects of underlying disease biology rather than a causal relationship. Prolonged induction therapy, even if with the same regimen, may also increase the risks of complications such as PI-related neuropathy, IMID-related financial toxicity, and the ‘time toxicity’ of additional time in clinic.

So what should clinicians do in this scenario? On the one hand, moving directly to ASCT after 4–6 cycles of induction runs the risk of undertreating some patients who might benefit from deeper responses upfront. On the other hand, delaying ASCT to pursue second-line induction runs the risk of overtreating some patients in the absence of a modern-era survival benefit. Given that MM therapies continue to improve in the relapsed setting, we conclude that the risks of overtreatment to ‘force’ a ≥VGPR with induction outweigh the risks of potential undertreatment. As such, we suggest proceeding directly to ASCT in patients who have achieved ≥PR with induction. In cases of MR as best response, proceeding directly to ASCT is reasonable for patients with low disease burden at baseline.

There are several nuances to these recommendations outside the scope of this Editorial. While we define an ‘insufficient’ response as ≤PR for the purposes of discussion, there is no clear consensus on what threshold defines such a response. In some cases, risk stratification based on bone marrow plasma cell burden or cytogenetic abnormalities may help with decision-making [9, 10]. Certain ASCT-related steps such as chemomobilization during stem cell collection or investigational conditioning (e.g., adding busulfan to melphalan) may potentially improve disease control, although their clinical benefit is not clearly established. Finally, every patient must be evaluated individually: unique factors like symptom burden, logistical considerations, and adherence to the original induction regimen may influence decision-making here.

In conclusion, there is no perfect approach for patients with insufficient responses to induction therapy. At the end of their hit song, the Clash states that “If I go, there will be trouble / And if I stay, it will be double.” While this twofold relative risk perhaps does not extrapolate perfectly, we agree that the benefits of going directly to ASCT generally outweigh the benefits of prolonging induction. In general, we suggest proceeding to ASCT rather than pursuing second-line therapy for patients who achieve a PR or better. Ultimately, the correct answer to this question is the one that works the best for the patient and their physician.
